# Molecular Origin of Strength and Stiffness in Bamboo Fibrils

**DOI:** 10.1038/srep11116

**Published:** 2015-06-08

**Authors:** Sina Youssefian, Nima Rahbar

**Affiliations:** 1 Department of Mechanical Engineering, Worcester Polytechnic Institute, Worcester, MA; 2 Civil and Environmental Engineering Department Worcester Polytechnic Institute, Worcester, MA

## Abstract

Bamboo, a fast-growing grass, has a higher strength-to-weight ratio than steel and concrete. The unique properties of bamboo come from the natural composite structure of fibers that consists mainly of cellulose microfibrils in a matrix of intertwined hemicellulose and lignin called lignin-carbohydrate complex (LCC). Here, we have used atomistic simulations to study the mechanical properties of and adhesive interactions between the materials in bamboo fibers. With this aim, we have developed molecular models of lignin, hemicellulose and LCC structures to study the elastic moduli and the adhesion energies between these materials and cellulose microfibril faces. Good agreement was observed between the simulation results and experimental data. It was also shown that the hemicellulose model has stronger mechanical properties than lignin while lignin exhibits greater tendency to adhere to cellulose microfibrils. The study suggests that the abundance of hydrogen bonds in hemicellulose chains is responsible for improving the mechanical behavior of LCC. The strong van der Waals forces between lignin molecules and cellulose microfibril is responsible for higher adhesion energy between LCC and cellulose microfibrils. We also found out that the amorphous regions of cellulose microfibrils are the weakest interfaces in bamboo fibrils. Hence, they determine the fibril strength.

Bamboo is the fastest growing naturally occurring bio-composite material^1^ that reaches maturity within months. As one of the well-known materials with high strength-to-weight ratios, bamboo has been used as renewable and sustainable structural material for decades^2^,^3^. The structure of bamboo consists of epidermis, parenchyma cells and vascular bundles, which are surrounded by supporting fibers. Researchers attribute the remarkable mechanical properties of bamboo to the presence of these fibers within the bamboo culm^4^,^5^. The unique mechanical properties of bamboo fibers come from their composite structure, in which cellulose fibrils are surrounded by a matrix of mainly lignin and hemicellulose^6^,^7^,^8^.

Prior researchers have investigated the mechanical properties of cellulose microfibers, lignin and hemicellulose and their interactions^9^,^10^,^11^,^12^,^13^,^14^,^15^,^16^,^17^,^18^,^19^,^20^. Among those previous efforts, Besombes and Mazeau^21^,^22^ performed molecular dynamics simulations on the assembly of a threo guaiacyl β-O-4 dimer of lignin and different surfaces of cellulose Iβ. They showed that the adsorption of lignin onto cellulose is surface-dependent. They also found a major contribution of van der Waals interactions onto (100) face and a major influence of hydrogen bond interactions in the adsorption of lignin onto (110) and (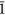
10) faces. Linder *et al.*^23^ performed non-equilibrium molecular dynamics simulations of lignin and cellulose. They found that lignin strongly associates with the cellulose microfibril. Nevertheless, few attempts have been made to use the crosslinked structure of lignin-hemicellulose to study the mechanical behavior of bamboo microfibrils and the interfaces of its matrix with cellulose microfibrils.

In this study, we investigate the role of each material in the mechanical properties of bamboo microfibrils as well as the underlying mechanisms of interactions between the matrix and cellulose microfibrils. With this aim, molecular models of lignin, bamboo hemicellulose and a crosslinked structure of these two materials as representative matrix materials were developed. Molecular dynamics techniques were used to elucidate the structures, thermodynamic and mechanical properties of the lignin, hemicellulose and the matrix. It is important to note that the properties of the systems under study are known to be sensitive to the percentage of water molecules^19^. Therefore, no water molecule was added to the simulations to remove this effect from the results and merely study the effects of the nanostructure on the thermodynamic and mechanical properties. Moreover, this assumption is not far from the actual bamboo culms that are currently exploited in industry because they undergo a heat treatment process to create a strong tub by removing their moisture.

## Molecular Structure of Bamboo Fibrils

**Figure 1:**
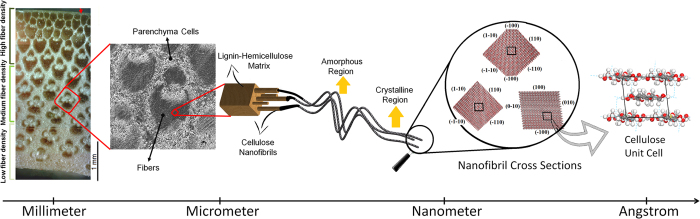
Hierarchical structure of bamboo. The vascular bundles in the parenchyma matrix are surrounded by supporting fibers which are known to be the source of remarkable mechanical properties of bamboo. Bamboo fibers have a hierarchical structure in which cellulose microfibrils reinforce the intertwined hemicellulose-lignin matrix. Linear chains of glucose with orderly hydrogen bonds form the crystalline regions of microfibrils while irregular hydrogen bonds create the amorphous regions. The cross section of these microfibrils is either rectangular or hexagonal.

Figure 1 presents the structure of bamboo fibers at different scales down to its building unit cell. The most abundant carbohydrate in bamboo fibrils is cellulose with the volumetric percentage around 73.83%. Cellulose microfibrils are formed by assembling linear chains of aldehyde sugars often referred to as glucose molecules, to make either rectangular or hexagonal cross sections with diameters of 3 to 5 nm^10^,^24^. If the hydrogen bonds between the hydroxyl groups form in an order, highly ordered (crystalline) regions are formed. However, if random hydrogen bonds form, disordered (amorphous) regions develop^10^,^25^. The positions of the hydroxyl groups determine the crystal system. These can be either triclinic or monoclinic unit cells (α or β type, respectively) with latter being the building block of plants such as bamboo. In a bamboo fiber, cellulose microfibrils are surrounded by lignin-carbohydrate complex (LCC) matrices that mainly contain lignin and hemicellulose with volumetric percentages of 10.50% and 12.49%, respectively.

Lignin is a natural phenolic macromolecule that mainly presents in the plant secondary cell wall. It is made up of three main phenylpropanoid sub-units, namely p-hydroxyphenyl (H-type), guaiacyl (G-type) and syringyl (S-type) units^26^. The biosynthesis of lignin occurs from different polymerizations of these three subunits. Hence, there are many possible bonding patterns between the individual units. Advancements in spectroscopic methods, however, have enabled scientists to elucidate the leading structural features of lignin^27^. They have also enabled scientists to propose different models for the molecular structure of lignin^28^,^29^,^30^,^31^,^32^. In this study, a structural model with 28 subunits of lignin proposed by Sakakibara (1980) has been used^33^. In this model, the value of the structural units and the number of protons per C_9_ structural units are close to that of spruce milled wood lignin reported by other researchers.

In order to be able to provide rigid support and shape to plants, lignin polyphenols are linked together in a three-dimensional crosslinked structures by covalently bonding to hemicellulose^34^,^35^,^36^. Hemicelluloses are a heterogeneous group of polysaccharides that unlike the cellulose, frequently have side chain groups. They are essentially amorphous with little strength^37^. The two major categories of hemicelluloses are glucomannans and xylans. Bamboo hemicellulose has been shown to be a xylan and further characterized as a β-(1→4)-linked-xylopyranosyl backbone, with the presence of L-arabinofuranose and 4-O-methyl-D-glucuronic acid as single side chains (4-O-methy1-D-glucurono- arabino-xylan^38^) that are arranged in an irregular manner. Therefore, the positions of the side chains are not fully determined. With the ratio of uronic acid/arabinose/xylose of 1:3:32 reported^39^, we consider hemicellulose structure as hybrid chains of two extreme positions of the side chains, one is a hemicellulose structure in which two functional groups are attached to the adjacent xylans and are in the closest possible distance (CPD). The other has the functional groups in the furthest possible distance (FPD). A preliminary study of the energy of different configurations revealed that the total energies were of the same order of magnitude. Hence, from the energy point of view, they are both acceptable. Hemicellulose molecules bond to lignin by a variety of different chemical bonds, however, most of the evidence refers to ether and ester bonds. Jeffries proposed structures for ester and ether linkages for lignin/uronic acid and lignin/arabinoxylan groups, respectively^40^. These linkage models have been used to create the bonds between lignin and hemicellulose in the crosslinked LCC network.

Based on the structures discussed here, molecular models of the typical lignin, hemicellulose, LCC and cellulose microfibrils were created. Atomistic simulation techniques were used to study the structure, thermodynamic and mechanical properties, and interactions of the bamboo fiber materials. The process of simulation is thoroughly described in the Method section.

## Results and Discussion

### Structure

**Figure 2:**
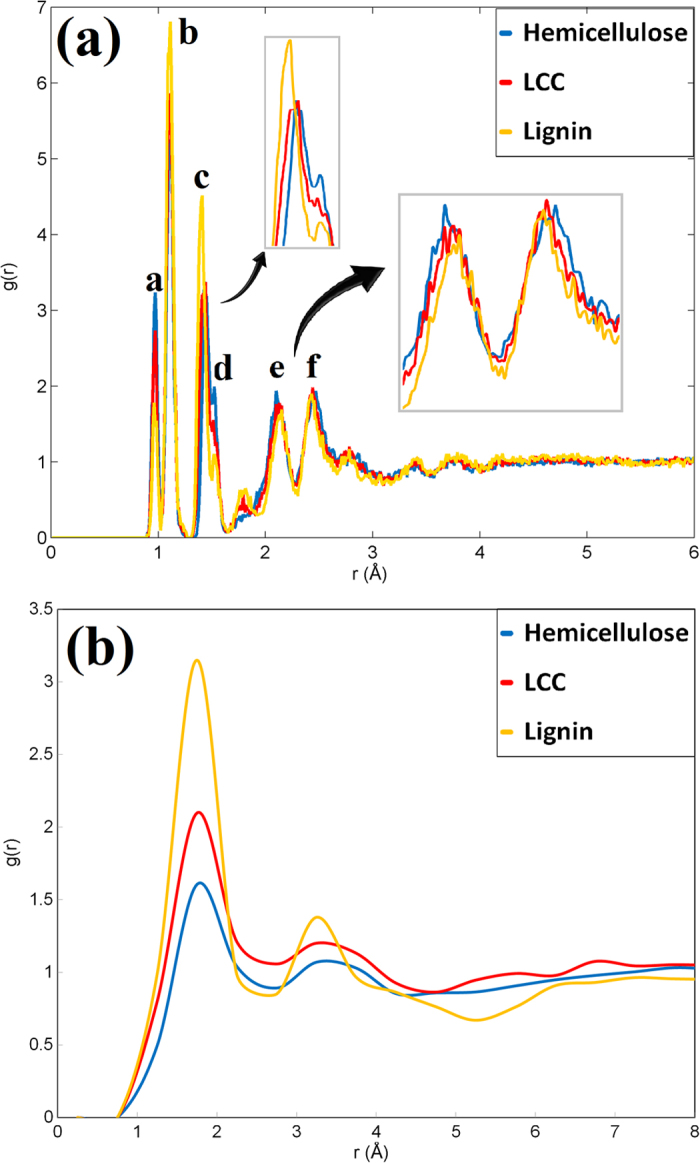
**a**) Radial distribution functions of all atoms in hemicellulose, LCC and lignin. The first four peaks, a; b; c and d, are related to covalent bonds of O−H, C−H, C−C and C−O, respectively. The fifth and sixth peaks, e and f, exist due to the non-bonded interactions in the systems. **b**) Radial distribution functions between hydrogen atoms of hydroxyl groups and the oxygen atoms in hemicellulose, LCC and lignin. The first peak at 1.85 Å is related to hydrogen bonds and the second peak at 3.25 Å is attributed to the oxygen-oxygen distance on two hydroxyl groups bonded by hydrogen bonds.

Radial Distribution Functions (RDF) of all atoms in lignin, LCC and hemicellulose structures are presented in Fig. 2a. According to this diagram, C−H (second peak, b) is the most abundant covalent bond in these materials. The abundance of the O−H (first peak, a), however, is different. For hemicellulose O−H has almost the same abundance as C−C, whereas for lignin, C−C is more abundant than O−H. Hence hemicellulose, presumably, is a better candidate than lignin for making hydrogen bonds. This is confirmed by the number of hydroxyl groups in the equal volumes of hemicellulose, LCC and lignin which are 1152, 784 and 456, respectively.

In Fig. 2a, the fifth peak is related to the distance between hydrogen and oxygen atoms, connected by hydrogen bonds, and the sixth peak is the distance between two non-bonded carbon atoms on the cyclohexane rings that are connected by a carbon atom (for example, carbon 1 and 3 on the ring). Comparing the abundance of hydrogen bonds (peak e) with the abundance of hydroxyl group (peak a), suggests that in the structure of lignin, most of the hydroxyl group hydrogens participate in hydrogen bonding, whereas in the structure of hemicellulose, the contribution of hydroxyl groups is about 60%.

To further understand the significance of hydrogen bonding, the RDFs between hydrogen atoms of hydroxyl groups and the oxygen atoms are obtained and presented in Fig. 2b. The first sharp peak at 1.85 Å is related to hydrogen bonding while the secondary broad peak at 3.25 Å is attributed to the oxygen-oxygen distance on two hydroxyl groups bonded by hydrogen bonds. The higher peak of lignin on the diagram confirms that greater numbers of lignin hydroxyl groups participate in hydrogen bonding than those in LCC and hemicellulose. This is because of the geometry of the functional groups, adjacent to these hydroxyl groups. The lignin hydroxyl groups are mostly parts of well-spread hydroxymethyl groups that are extended out from the main chains. The hemicellulose hydroxyl groups, however, are parts of xylose groups, where two hydroxyl groups attach directly to the main chains and are localized on pyranose rings. Hence, lignin hydroxyl groups are more exposed to and more accessible by other hydroxyl groups than those of hemicellulose hydroxyl groups. Therefore, although the hemicellulose structure contains more hydroxyl groups, lignin hydroxyl groups are more efficient in making hydrogen bonds.

The hydrogen bond analysis from the geometry of the materials showed that the average length of hydrogen bonds for all three materials is around 1.88 Å (ranging from 1.58 Å to 2.49 Å) and the average angle of donor-hydrogen-acceptor is around 150.96° (ranging from 120° to 180°). This is associated with short and strong hydrogen bond interactions. Such strong hydrogen bonds come from the existence of cooperativity. Cooperativity or cooperative effect between hydrogen bonds implies that the hydrogen atom of a hydroxyl group can form a stronger hydrogen bond, if the oxygen creates a hydrogen bond with an adjacent hydroxyl group^41^. This phenomenon is observed in most of the hydrogen bonds in the three materials.

### Thermodynamic properties

Density and glass transition temperature are good physical properties for evaluating the realism of the conformations. The computed glass transition temperature for LCC, presented in Table 1, falls between the obtained values for hemicellulose and lignin which are in good agreements with the experimental data. The final relaxed conformations of lignin and hemicellulose have average densities of 1.26 ± 0.02 g/cc and 1.45 ± 0.03 g/cc, respectively. These are comparable with experimental results, in Table 2. Hence the lignin and hemicellulose models estimate the real densities within 5.2% and 4.6%, respectively. These differences are partly attributed to the vacuum condition assumed in the simulation that does not occur in the experiments.

The density of LCC relaxed conformation was calculated to be 1.34 ± 0.02 g/cc. Implementing the simple rule of mixture density, 
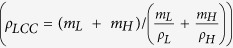
, in which two materials are mixed physically without any changes in mass or volume, the LCC density, 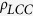, was estimated to be about 1.35 g/cc, using densities obtained from the lignin and hemicellulose simulations. This value is close to the density of the relaxed LCC model, suggesting that the volume fractions of hemicellulose and lignin do not significantly change in the process of LCC creation. Hence, the true value of LCC density can be estimated at 1.41 g/cc using experimental values of lignin and hemicellulose densities.

### Mechanical properties

The Young’s moduli of the hemicellulose, lignin and LCC are presented in Table 3. The average Young’s moduli of lignin and hemicellulose were estimated to be 5.90 ± 0.37 GPa and 8.40 ± 0.15 GPa, respectively. These are in good agreement with the respective experimental measurements of 6.7 GPa and 8.0 GPa for nearly dry Pinus radiata lignin and hemicellulose (consisting of arabino-4-O-methylglucuronoxylan which have a close structure to bamboo hemicellulose)^14^,^15^. Other studies suggest respective values in the range of 1 to 2 GPa and 3.5 to 7 GPa for different types of lignin and hemicellulose^16^,^17^. Hence, the results from the simulations and experiments show that hemicellulose has better mechanical properties than lignin. Performing the same procedure on the LCC model resulted in an average Young’s modulus of 6.93 ± 0.31 GPa, which is between the Young’s modulus of hemicellulose and lignin.

The Young’s modulus of a material is defined by its resistance to the tension or compression of atoms and molecules. It is affected by factors such as interatomic and intermolecular energies per unit volume. For amorphous materials, such as LCC, lignin and hemicellulose, applied stresses are mostly used to overcome the non-bonded energies between the molecules because they unwind the chains rather than directly struggle with the energies of bonds between the atoms. Therefore, at small strains, non-bonded energies play more important roles in determining the elastic moduli of amorphous materials.

**Figure 3:**
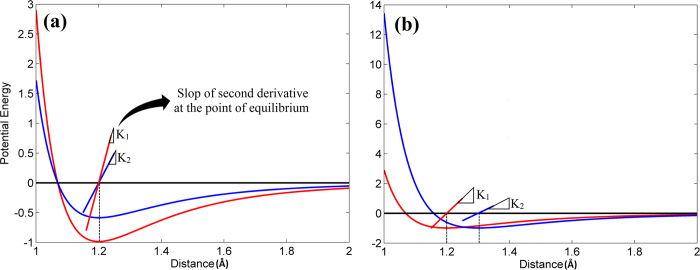
**a**) When the potential energies have the same equilibrium distance, the potential with a lower well depth exhibits a higher stiffness between two atoms and **b**) When the potential energies have the same well depth, the potential with a shorter equilibrium distance corresponds to a higher stiffness between two atoms.

However, the effects of non-bonded energies are not equal. According to the Lennard-Jones function, non-bonded energies with lower equilibrium energies or shorter equilibrium distances exhibit greater resistance to stretching or compressing. Thus, they have higher stiffness. Figure 3a illustrates that a lower minimum potential energy at the point of equilibrium corresponds to a greater curvature (second derivative) of the energy- distance curve. This curvature is equal to the force - distance slope (stiffness). Therefore, a lower minimum potential energy creates a greater stiffness. The same situation holds true for the case where the minimum energies are the same but equilibrium distances are different (Fig. 3b). In this case, the shorter equilibrium distance has a greater curvature and thus a greater stiffness. Therefore, the strong short-range hydrogen bonds that have shorter equilibrium distance with lower minimum potential energy exhibit higher stiffness and play more important roles in determining the Young’s modulus than the other weak long-range non-bonded interactions.

An estimation of hydrogen bond stiffness along the line between the hydrogen and acceptor can be derived from a simple Lennard-Jones potential energy function (Equation 1). A more complete expression that includes the angle dependence of hydrogen bonds can be used in the future to calculate the accurate stiffness.


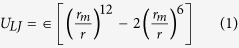


where r is the distance between the two atoms, ϵ is the depth of the potential well and r_m_ is the equilibrium distance. The forces between the two atoms can be calculated from the derivative of the potential energy, 
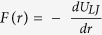
.


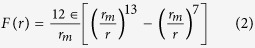


The stiffness that this force causes around the equilibrium point is obtained from the derivative of the force, 
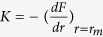
, which results in,


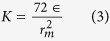


For a hydrogen bond between two hydroxyl groups, the equilibrium distance was calculated to be around 1.85 × 10^−10^ m, and the potential depth is reported to be 1.2 × 10^−20^ J^42^. With these values a rough estimation of stiffness of a hydrogen bond in hemicellulose, LCC and lignin was found to be around 25.24 J/m^2^. Since hydrogen bonds bear the stresses in the system, more hydrogen bonds connect more atoms and create a stiffer system. That is why hemicellulose with a 1.08 × 10^8 ^J/m^3^ hydrogen bond energy density has the highest Young’s modulus among the three materials while LCC with a hydrogen bond energy density of 7.51 × 10^7 ^J/m^3^ has a higher Young’s modulus than lignin with a 4.46 × 10^7^ J/m^3^ hydrogen bond energy density.

### Adhesive interactions

**Figure 4:**
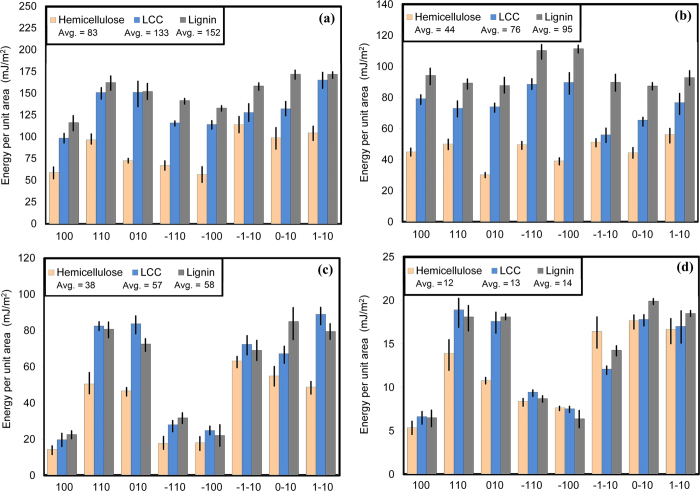
**a**) The adhesion energy per unit area between different cellulose microfibril faces and hemicellulose, LCC and lignin. The average energy between lignin molecules and a cellulose microfibril is higher than the energy between hemicellulose and cellulose microfibrils. **b**) The van der Waals energy per unit area between different cellulose microfibril faces and hemicellulose, LCC and lignin. Lignin exhibits higher adhesive energy to cellulose microfibrils than hemicellulose. **c**) The electrostatic energy per unit area between different cellulose microfibril faces and hemicellulose, LCC and lignin. The average electrostatic energy between lignin molecules and cellulose microfibrils exhibit no significant difference from the electrostatic energy between hemicellulose and cellulose microfibrils **d**) the hydrogen bond energy per unit area between different cellulose microfibril faces and hemicellulose, LCC and lignin. The average hydrogen bond energies between cellulose microfibrils and the three materials are similar.

In the composite structure of a bamboo fiber, knowledge of adhesive interactions between the different layers determines its strength. Applied stresses on a microfibril are carried either by cellulose microfibrils, the LCC matrix or the interfaces of these two regions. To investigate the adhesion energies at these interfaces, twenty-four different assemblies of lignin, hemicellulose and LCC on top of cellulose substrates were created, each of which simulates the interaction between one of the materials and one face of the eight possible faces of cellulose microfibrils. The adhesion energies were computed from final trajectories of the simulations and presented in Fig. 4a. Although these results indicate that the overall adhesion energies for these materials are different, their tendencies to adhere to microfibril faces exhibit almost the same pattern. For each material, the interaction energies of (100) and (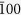) faces are the lowest whereas the energies of other faces vary around an average value. The average adhesion energy between lignin and microfibril faces was about 152 mJ/m^2^. This is higher than adhesion energy between LCC and microfibril faces which is about 133 mJ/m^2^. Hemicellulose with average adhesion energy of around 83 mJ/m^2^ shows the lowest adherence to microfibril among the three materials. This adhesion trend, also, has been shown by Hosoya *et al.* in the pyrolysis of hemicellulose and lignin with cellulose where lignin-cellulose interactions were significant compare to low hemicellulose-cellulose interactions^43^. Therefore, lignin with greater overall adhesion energy to cellulose is responsible for providing strong interaction between LCC matrix and cellulose microfibrils to create strong bamboo fibrils. To understand the mechanism of interactions between these materials and cellulose, we computed the electrostatic and van der Waals energies, accountable for the adhesion, as shown in Fig. 4b,c. These results suggest that the van der Waals energies do not change significantly over the microfibril faces whereas the electrostatic energies of (100) and (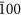) faces are less than that of other faces. Hence, the electrostatic energy is responsible for reduction of adhesion energy between cellulose (100) or (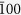) faces and hemicellulose, LCC and lignin. The average electrostatic energies between cellulose microfibril faces and hemicellulose, LCC and lignin are 38 mJ/m^2^, 57 mJ/m^2^ and 58 mJ/m^2^, respectively, and the average van der Waals energies between cellulose microfibril faces hemicellulose, LCC and lignin are 44 mJ/m^2^, 76 mJ/m^2^ and 95 mJ/m^2^, respectively. It is evident that lignin van der Waals energy is higher around 116% than that of hemicellulose whereas the electrostatic energy are higher just by about 50%. This indicates that the superiority of lignin adhesion energies to cellulose comes from the relatively higher van der Waals energies between cellulose microfibril and lignin. One of the major components of electrostatic energies at the interface of cellulose microfibril and hemicellulose, LCC and lignin, is hydrogen bonding which are illustrated in Fig. 4d. Regardless of the slightly higher average hydrogen bond energy between lignin and cellulose, all three materials have almost the same hydrogen bond interaction energies with cellulose microfibrils. The hydrogen bond energies show a similar pattern to the electrostatic energies presented in Fig. 4c. In other words, (100) and (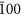) faces have the lowest hydrogen bond energies which are similar to that of the electrostatic interactions. This suggests that different level of hydrogen bond energies at the interface of microfibril and hemicellulose, LCC and lignin are the main reason for different electrostatic energies from one face to another, causing the adhesion energies between the matrix and (100) and (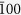) faces of microfibril to drop.

**Figure 5:**
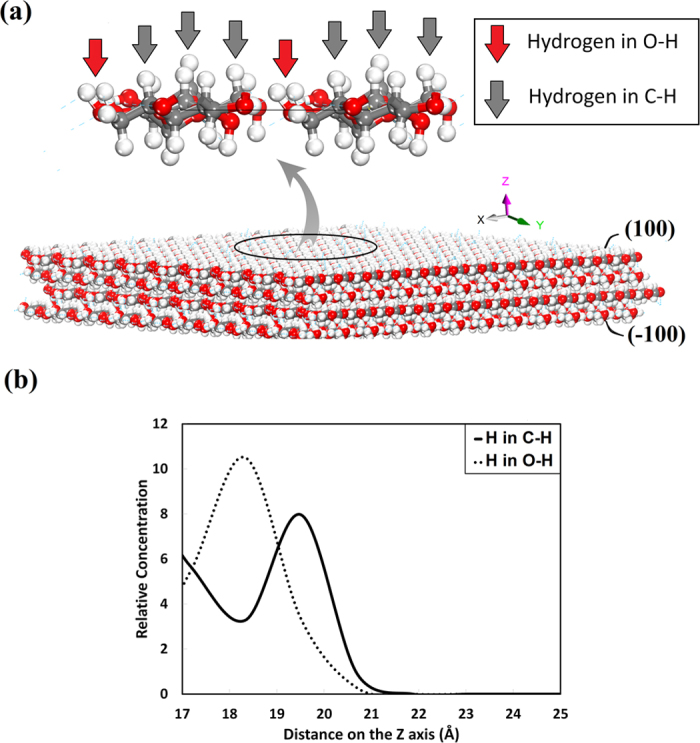
**a**) Distribution of hydrogen atoms on the (100) and (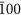) surface. These surfaces are covered with hydrogen atoms that are bonded either to oxygen or carbon. The hydrogen atoms that are connected to oxygen are less exposed than the hydrogen atoms connected to carbon because they stay closer to the surface. **b**) The relative concentration of hydrogen in O−H and C−H along Z axis. Hydrogen in O−H groups accumulates at 18.3 Å, while the hydrogen in C−H groups accumulates at 19.8 Å.

A closer look at the (100) and (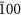) face provides some insights into the weak hydrogen bonds between the faces and the matrix. Figure 5a shows that the surfaces of (100) and (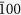) are covered with hydrogen atoms (white spheres). The hydrogen atoms are bonded either to oxygen or carbon. The hydrogen atoms connected to oxygen are less exposed because they stay closer to the surface while the hydrogen atoms connected to carbon face outwards and are configured for contact with adjacent layers. Therefore, since the accessibility of the hydrogen atoms in O−H groups is less than that in C−H groups, the overall hydrogen bond energy between these two faces and the matrix diminishes. Figure 5b confirms this observation by comparing the relative concentration of hydrogen atoms that are attached to carbon and oxygen. On this diagram, the relative concentration of the top layer of hydrogen on O−H groups and C−H groups are illustrated along the Z axis. Hydrogen in O−H groups accumulates at 18.3 Å, while the hydrogen in C−H groups accumulates at 19.8 Å. Therefore, the latter group is more exposed to other layers.

**Figure 6:**
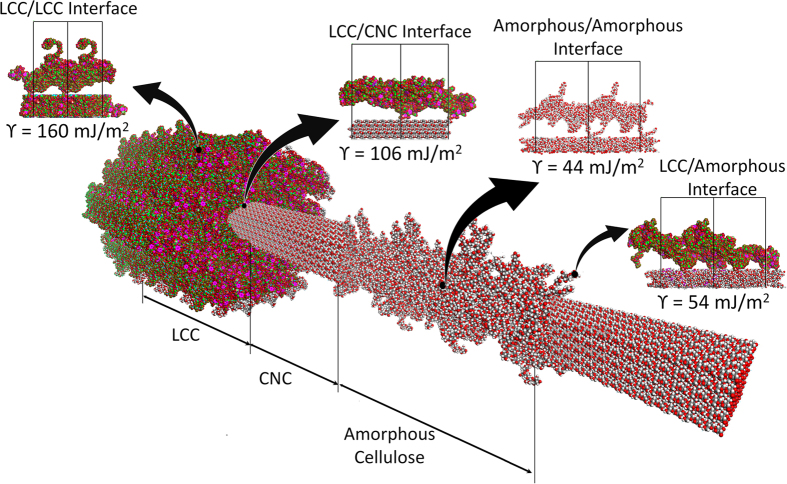
The adhesion energy per unit area between different interfaces, present in a possible nanostructure of bamboo fiber. The adhesive interaction energy at the interface of LCC layers is the highest among all the regions. The amorphous regions exhibit the lowest adhesive interactions, hence, their interface strength are likely to determine the strength of overall strength of bamboo fibrils.

This adhesion study proves that the weakest interaction between cellulose microfibril and LCC occurs at the (100) and (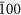) faces. However, the question of which interface is the weakest link in a microfibril still remains. To answer this question, we need to calculate the other possible locations where defects may occur. Figure 6 shows a schematic of a microfibril in a matrix of LCC that is woven around both crystalline and amorphous regions. In this structure, the stress can detach either layers of LCC/LCC, interfaces of LCC/crystalline cellulose, LCC/amorphous cellulose, or the stress can fracture the cellulose microfibril.

The weakest adhesion energy between cellulose microfibrils and LCC was found to be around 106 ± 8 mJ/m^2^. This energy is less than that between the two layers of LCC in the matrix, which was estimated at about 160 ± 29 mJ/m^2^. The adhesion energy between LCC and the amorphous region of microfibrils was close to the adhesion energy of amorphous cellulose/amorphous cellulose interface which were estimated to be 54 ± 10 mJ/m^2^ and 44 ± 6 mJ/m^2^, respectively. Therefore, the amorphous region of microfibril has the lowest adhesion energy in the system.

**Conclusion.** In this study, atomistic simulation techniques have been used to investigate the nanoscale mechanical properties of bamboo fibrils. In particular, the role of the two major components of bamboo LCC (hemicellulose and lignin), in the remarkable properties of bamboo fibrils, was investigated. The simulations of the density and Young’s modulus resulted in predictions that were in good agreements with available experimental data. Hemicellulose was found to improve the thermodynamic and mechanical properties of the matrix whereas lignin was found to improve the adhesion between the matrix and the cellulose microfibrils. The LCC mechanical properties and the adhesion energies were found to be between those of hemicellulose and lignin. The superiority of hemicellulose’s mechanical properties is due to the large number of hydroxyl groups, that increases the hydrogen bond energy density. Lignin strong adherence to cellulose microfibrils comes essentially from the large van der Waals energies between lignin and cellulose. The adhesion energy varies over the microfibril faces. For the (100) and (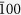) faces it was found to be the lowest due to the low hydrogen bond energy between the microfibrils and the matrix. Comparing the results of the adhesion energies of other adjacent layers in the bamboo fibrils revealed that the interface of the LCC and amorphous region of cellulose microfibrils is the weakest link in the system. It is, therefore, likely to determine the lower bound strength of bamboo fibrils.

## Methods

COMPASS (cff91 ver. 2.6^44^) was chosen as a proper force field for atomistic simulations of carbohydrate. This force field is one of the first ab-initio based force field that can capture the structural, thermal and mechanical properties of vast range of molecules and atoms including cellulose^45^,^46^,^47^,^48^. In COMPASS, the non-bonded energies include van der Waals and electrostatic energies, with hydrogen bonds being a natural consequence of electrostatic energies. The non-bonded interactions are described by the summation of the Lennard-Jones (LJ) function for van der Waals energies, E_VdW_, and Columbic representation for electrostatic energies, E_Ele_. Combination of Coulombic and LJ terms has been shown to treat hydrogen bonding with reasonable accuracy, including the angular dependencies. Therefore, COMPASS force field can accurately predict structural and conformational properties for a broad range of molecules including carbohydrates, without considering any specific terms for hydrogen bonds. Studies on hydrogen bonds were conducted on the final structures with the following criteria:

1. The maximum distance between the hydrogen and the acceptor atom for which hydrogen bonding is possible is 2.5 Å.
2. The minimum angle between the donor, hydrogen and acceptor atoms in degrees for which hydrogen bonding is possible was chosen as 120°.

For calculating the hydrogen bond energy, we have used a CHARMM-like hydrogen bonding potential such as Equation (4),


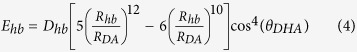


where *θ*_*DHA*_ is the bond angle between hydrogen donor (*D*) and the hydrogen (*H*) and the hydrogen acceptor (*A*). R_DA_ is the distance between the donor and acceptor. The values of D_hb_ and R_hb_ were adopted from the literature^49^.

### Molecular modeling

A monoclinic unit cell with a symmetry group of P2_1_ and parameters reported by Nishiyama *et al.*^50^ were used to create a unit cell of cellulose. This model was optimized using a Smart algorithm, which is a cascade of the steepest descent, adjusted basis set Newton-Raphson (ABNR), and quasi-Newton method. An NPT dynamic simulation at a temperature higher than cellulose melting point (700 K for 50 ps) was performed, followed by an NPT at the room temperature for 50 ps.

The cellulose cell parameters estimated by COMPASS are a = 7.797 Å, b = 8.210 Å, c = 10.397 Å and γ = 96.5° and the estimated mechanical properties are E_X_ = 16.24 GPa, E_Y_ = 56.56 GPa and E_Z_ = 125.89 GPa with a density of 1.66 g/cc. The reported experimental data for mechanical properties of cellulose are E_X_ = 11 − 57 GPa, E_Y_ = 50 − 57 GPa and E_Z_ = 90 − 200 GPa and the density is 1.65 g/cc^9^,^10^,^11^,^51^,^52^. The assessed model, then, is cleaved on (100), (110), (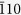), (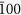), (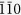) and (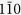) surfaces that represent the six sides of hexagonal cross section, along with (010) and (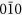) surfaces that represent the two remaining sides of the rectangular cross section. Eight vacuum slabs with sizes of around ~ 5 nm × ~ 5 nm and a thickness of about ~2.5 nm were created from the equilibrated surfaces.

**Figure 7:**
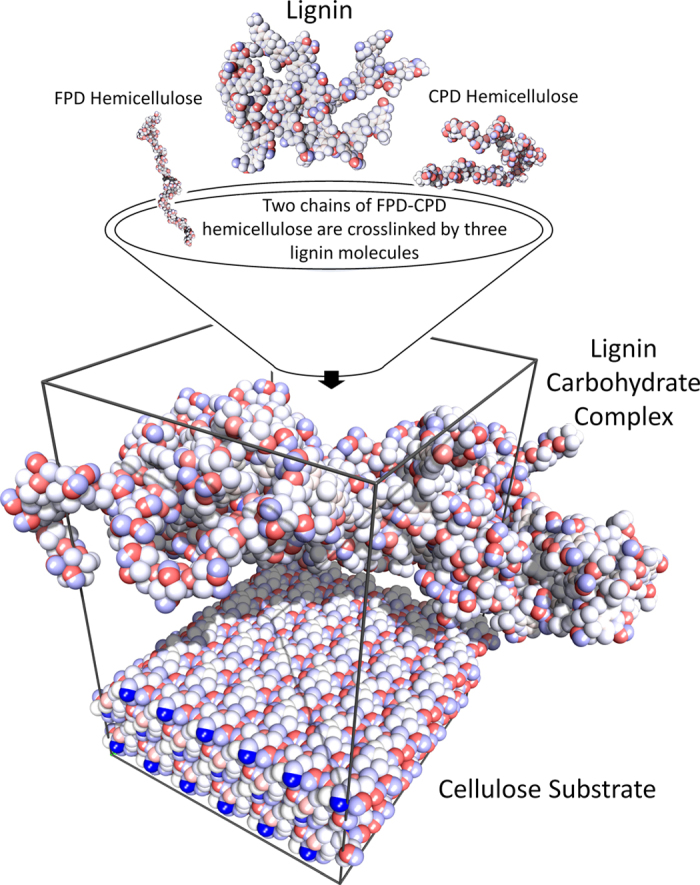
Process of preparing LCC models and atomistic simulation. The hemicellulose chain was created from one chain of CPD and one chain of FPD. Four hemicellulose chains were randomly crosslinked by three lignin molecules to create an LCC structure. Lignin, hemicellulose and LCC models were placed on amorphous cellulose and eight substrates of crystalline cellulose which are representing eight possible faces of microfibrils. The NVT dynamic simulations at 300 K with 1 fs time step were performed for 1.2 ns and the adhesion energies were calculated from the final trajectories.

The dimensions of the system should be large enough to statistically capture all possible local realizations of the structures. Since this is computationally expensive, the simulations had to be conducted on multiple smaller independently prepared polymers. Hence, three different models of each material were chosen for all the simulations. The final results are the average of all three configurations. The simulation process, schematically presented in Fig. 7, starts with modeling lignin, hemicellulose and LCC. For the lignin models, eight lignin molecules, created from the structure described earlier, were packed into periodic boundaries. For the hemicellulose models, four hemicellulose chains, created by combining one chain of CPD with one chain of FPD, were packed into periodic boundaries. To create LCC structures, the correct number of mixing chains of the bamboo components should be calculated. Considering the ratio of lignin volumetric content to hemicellulose (r_LH _~ 1.0), their densities (ρ_L_ = 1.33 g/cc^53^,^54^ and ρ_H_ = 1.52 g/cc^55^, respectively) and molecular masses (M_L_ = 5149.40 g/mol and M_H_ = 4816.19 g/mol, respectively) in bamboo structures, the ratio of number of lignin units (N_L_) to hemicellulose units (N_H_) was found to be 0.8 (using Equation 5). Hence, three lignin molecules were randomly cross-linked with four hemicellulose chains to create a model of bamboo LCC with the specific ratio.


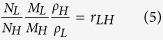


To search for the polymer configurations with the lowest energies, a metaheuristic algorithm was used for locating a good approximation of the global minimum, by simulated annealing^56^. The results of the annealing process were relaxed by a series of NVT and NPT dynamic simulations, according to the method in ref. [57], to achieve equilibrium in the system without any artificial energy resulted from the previous processes. At the end of the relaxation process, the structure was acceptable for further simulations, if it could predict the density correctly. The results of this process are hemicellulose, LCC and lignin periodic structures which represent realistic conformations of these materials with sizes of 5.34 × 5.34 × 5.34 nm^3^, 5.41 × 5.41 × 5.41 nm^3^ and 5.45 × 5.45 × 5.45 nm^3^, respectively.

### Structure

To study the nanostructures of these models, a Radial Distribution Function (RDF) was used. RDF gives a measure of the probability of finding an atom, within a spherical shell of infinitesimal thickness at a distance, r, from the reference atom. Hansen and McDonald define the resulting function, g(r), as^58^:


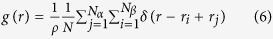


where N is the total number of atoms, and ρ is the overall number density and Nα and Nβ are the number of atoms of type α and β, respectively. RDF of all atoms in equilibrated lignin, LCC, hemicellulose, presented in Fig. 2a, was used to study the type and abundance of short and long range interactions. The identical positions of the peaks on the RDF diagram indicate that hemicellulose, LCC and lignin exhibit the same type of short and long range interactions. The different heights of the peaks, however, indicate differences in their structures, which are responsible for the diverse properties of these materials. The first four peaks, a, b, c and d, are related to covalent bonds O−H, C−H, C−C and C−O, respectively. The fifth and sixth peaks, e and f, exist due to the non-bonded interactions in the systems.

### Young’s modulus

The periodic structures were expanded along each direction to the maximum strain amplitude of 0.01 in 10 steps. In each step the stresses were obtained from virial stress expression which is commonly used to relate the computed stress in molecular dynamics to continuum stresses (The structures that were dependent on the maximum strain amplitude were eliminated from the results).

### Glass transition temperature

The glass transition temperature can be obtained from the change in the slope of specific volume-temperature curve^59^,^60^. To achieve this aim, the temperature of each system was increased to 700 K and slowly brought down to 100 K at the rate of 0.5 K/ps while the temperature and pressure were controlled by the Nose thermostat and Berendsen barostat, respectively. In 48 random steps, the system was equilibrated with NPT dynamics for 25 ps and the results were recorded to create the specific volume-temperature curves. These curves were used to compute the glass transition temperatures of hemicellulose, LCC and lignin (The structures that were dependent on the upper and lower temperature boundaries were eliminated from the results).

### Interactions

To investigate the interaction energies at the interfaces between cellulose microfibrils and hemicellulose, LCC and lignin, twenty-four different assemblies of lignin, hemicellulose and LCC on top of cellulose substrates were created, each of which simulates the interaction between one of the materials and one face of the eight possible faces of cellulose microfibrils. The cellulose substrates were fixed in all directions whereas the top layers were free to move during the simulations. The size of the moving layers determines the simulation time span, given by the time required for the atoms to travel towards the substrate and to reach the steady state. Hence, the simulation time is regulated by diffusion properties of moving layers and can be consequently calculated by using the Strokes-Einstein equation. This equation estimates the diffusion constant of a small particle with a radius of 5 nm to be in the order of 10 −10 m^2^/s. Since the average traveling distance of moving layer is about 0.5 nm, simulation time is estimated to be about 1.2 ns 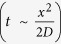. In this time period, the NVT dynamic simulations at 300 K with 1 fs time step were performed for three different conformations of these materials to minimize the effect of initial conformation on the final results. The adhesion energies were calculated from the simulation trajectories by subtracting the cellulose substrate energy (E_sub_) and the matrix energy (E_mat_) from the total energy (E_tot_) of each assembly (E_adh _= E_tot_ − (E_mat_ + E_sub_)). Assemblies of LCC/LCC, LCC/amorphous cellulose and amorphous cellulose/amorphous cellulose layers were modeled in NVT dynamic simulations at 300 K for 1.2 ns and the interaction energies (E_adh_) were computed.

## Additional Information

**How to cite this article**: Youssefian, S. and Rahbar, N. Molecular Origin of Strength and Stiffness in Bamboo Fibrils. *Sci. Rep.* **5**, 11116; doi: 10.1038/srep11116 (2015).

## Figures and Tables

**Table 1:** Glass transition temperature (°C) of lignin, hemicellulose and LCC obtained from the molecular calculations and experiments.

**Material**	**Simulation**	**Experiment**
Lignin	140.26	97-171
Hemicellulose	186.06	140-180
LCC	166.11	N/A

**Table 2:** Density (g/cc) of lignin, hemicellulose and LCC obtained from the molecular calculations and experiments.

**Material**	**Simulation**	**Experiment**
Lignin	1.26	1.33
Hemicellulose	1.45	1.52
LCC	1.34	N/A

**Table 3:** Young’s Modulus (GPa) of lignin, hemicellulose and LCC obtained from the molecular calculations and experiments.

**Material**	**Simulation**	**Experiment**
Lignin	5.90	2 – 6.7
Hemicellulose	8.40	3.5 – 8.0
LCC	6.93	N/A
